# Community-based HIV testing in The Netherlands: experiences of lay providers and end users at a rapid HIV test checkpoint

**DOI:** 10.1186/s12981-021-00357-9

**Published:** 2021-06-23

**Authors:** Nori Krabbenborg, Ralph Spijker, Anna Maria Żakowicz, Milo de Moraes, Titia Heijman, Eline Op de Coul

**Affiliations:** 1 Center for Infectious Disease Control, National Institute for Public Health and the Environment (RIVM), P.O. Box 1, 3720 BA Bilthoven, The Netherlands; 2 STI Aids Netherlands, Amsterdam, The Netherlands; 3 Aids Healthcare Foundation (AHF) Checkpoint, Amsterdam, The Netherlands; 4 Public Health Service of Amsterdam, Amsterdam, The Netherlands

**Keywords:** HIV testing, Lay providers, Community approach, Key populations, MSM, LGBTQ, Asylum seeker, Migrant populations

## Abstract

**Background:**

The AIDS Healthcare Foundation (AHF-Checkpoint) in The Netherlands offers rapid HIV testing for key populations by lay providers. We explored the experiences and needs of lay providers and end users of HIV testing at AHF-Checkpoint, taking into account the WHO 5C-(consent, confidentiality, counselling, correct-results, connection-to-care) criteria for HIV test services.

**Methods:**

Qualitative evaluation with 15 semi-structured interviews conducted during 2020 with ten lay providers and five gay or bisexual end users. Recorded interviews were thematically analysed, taking data triangulation into account.

**Results:**

Four domains were identified: (1) accessibility of HIV testing, (2) quality of test procedures, (3) bridging (transitional care), and (4) future strategies for service delivery. AHF-Checkpoint fills a gap for key populations including LGBTQ and refugees, who experience HIV testing barriers at sexual health centres or general practices. The level of trust between lay providers and end users was highly valued by end users. They appreciated the low threshold to test at no costs, and the absence of waiting lists or triaging. Needs expressed by lay providers included more preparedness for emotionally charged situations, and extra training to improve STI knowledge. End users expressed a need for a full STI test package. Of the 5Cs, consent, counselling, and correct results were realised but confidentiality was sometimes difficult to achieve at pop-up locations, and referral barriers for confirmation testing (connection-to-care) were occasionally experienced by lay providers during weekends.

**Conclusion:**

AHF-Checkpoint was described as a convenient and easily accessible service by end users and lay providers. Of the WHO 5Cs, connection-to-care could be optimised to ensure HIV confirmation and STI testing through a liaison approach with professionals from the regular healthcare sector.

## Introduction

Despite the great successes that are globally achieved in HIV prevention and treatment, reaching people with undiagnosed HIV remains a challenge in many countries [1]. By 2025, the 95–95–95 targets (i.e. 95% diagnosed, 95% on antiretroviral (ART) treatment and 95% virally suppressed), as set by the Joint United Nations Programme on HIV/AIDS (UNAIDS), should be achieved [2]. By 2019, this HIV care continuum was 93–93-96 for The Netherlands [3]. Tracing the remaining 7% of people with undiagnosed HIV that corresponds to an estimated number of 1730 individuals, and linking them to care is part of the Dutch national action plan on STIs, HIV, and sexual health for 2017–2022 [4].

The main healthcare providers for HIV testing in The Netherlands are the public sexual health centres (SHCs), general practitioners (GPs), and hospitals. SHCs offer free (anonymous) STI- and HIV testing to specific risk groups including men who have sex with men (MSM), young people, people originating from STI and HIV endemic regions [5], and those who are notified for STI. HIV testing at the GP is not anonymous and may incur costs as part of the compulsory financial contribution [6].

Testing for HIV can be challenging for key populations due to barriers on psychosocial, cultural, religious or other grounds [7]. Barriers to testing reported by MSM generally fall into two groups: (1) structural barriers such as clinic opening hours, returning for results, or test costs, and (2) psychosocial barriers such as fear of an HIV positive result, fear of stigma and discrimination, and risk perception [8, 9]. Other key populations including persons with a migration status, female sex workers, and transgender women, experience similar barriers [10–12].

Community-based HIV testing by lay providers (trained non-medical staff) in non-healthcare settings is a potentially effective approach to help countries reach the 90–90–90 or 95–95–95 targets [13, 14]. In 2015, the World Health Organization (WHO) recommended HIV testing by lay providers as an additional strategy to reach key populations who may not test otherwise [15]. A systematic review on HIV testing in non-healthcare settings in European Union (EU) and European Economic Area (EEA) member states showed that testing in community- and outreach settings is effective in reaching people who have not previously tested for HIV, but a variety of findings on uptake and HIV positivity were found. Furthermore, self-sampling and self-testing were considered highly acceptable by key populations and same-day confirmatory testing in community settings was feasible [16]. Working with lay providers, who can be members of key populations, may help to discuss sexuality openly, avoid medical jargon, and reduce HIV- or risk group-associated stigma [17]. Thus, community-based HIV testing may increase access to key populations by offering low-threshold counselling, rapid testing, and connection-to-care by lay providers at convenient onsite (e.g. Checkpoints) or offsite locations (e.g. gay saunas, and cultural or social events).

HIV test services by lay providers are limited in the Netherlands. One of the initiatives is the AIDS Healthcare Foundation (AHF)-Checkpoint in Amsterdam. This low-threshold venue that provides rapid HIV testing is designed to counteract testing barriers for lesbian, gay, bisexual, transgender and queer (LGBTQ) populations, refugees and migrants from HIV endemic countries or anyone else who needs it. The foundation offers confidential, free ‘walk-in’ HIV counselling and testing in a safe, non-judgmental, environment with same-day results, referral to STI test facilities, and linkage to healthcare in case of a reactive test result [18, 19]. Lay providers receive theoretical training at AHF-Checkpoint in HIV/STI and practical training in performing counselling and rapid HIV testing, which includes motivational interviewing, shadowing experienced lay providers, individual test performance and feedback. Besides an onsite test location in Amsterdam, AHF-Checkpoint operates at four pop-up venues: Checkpoint Rotterdam (migrant populations and MSM), Checkpoint NZ (‘Nieuwezijds’) Sauna (MSM), Checkpoint Club Church (MSM) and Checkpoint Secret Garden (LGBTQ + migrant populations).

In 2018, AHF-Checkpoint tested 4708 people of whom 2989 (63%) were tested onsite in Amsterdam and 1719 (37%) were tested at pop-up venues in The Netherlands [19]. Of the persons tested, 61% were non-Dutch and 47% identified as MSM. The overall positivity rate was 0.8% (n = 37), slightly lower than in the previous three years (1.0–1.2%) [18]. HIV positivity among MSM was 1.4% in 2018, which is higher than the HIV positivity among MSM (0.5%) who tested at the Dutch SHCs in the same year [20]. In total, 81% of the HIV positive clients who tested at AHF-Checkpoint were successfully linked to care. People who are not referred are usually tourists or expats who may prefer follow-up in their country of origin [19].

There is a lack of insight in the experiences with rapid HIV testing by lay providers in The Netherlands. Therefore, we conducted a qualitative study including lay providers’ and end users’ perspectives on and experiences with HIV testing at AHF-Checkpoint. In this evaluation, their views were placed on the WHO 5Cs principle of the rights-based response to HIV: Consent, Confidentiality, Counselling, Correct test results, and Connections-to-care, treatment and prevention services [21, 22]. With the findings recommendations will be made to improve this community-based HIV test service in The Netherlands.

## Methods

### Design

This qualitative study was conducted at the National Institute for Public Health and the Environment (RIVM), in collaboration with AHF-Checkpoint, the public health service (PHS) in Amsterdam and STI Aids Netherlands. For the semi-structured in-depth interviews, two topic lists were developed: one for lay providers and one for end users. They were based on scientific articles and grey literature including international HIV testing guidelines from the WHO. The topic lists were reviewed by a multidisciplinary team including an epidemiologist, sociologist, and anthropologist. Open-ended example questions for each topic were developed to elaborate on issues that were considered important, such as the WHO 5C’s that embodies that all HIV test services should include pre-test information, post-test counselling, accurate HIV testing and diagnosis, and linkage to HIV prevention, care and treatment services [22].

### Study participants

Lay providers and end users were eligible for study participation if they were 18 years or older and able to understand Dutch or English. The recruitment of end users was restricted to MSM, as they were the majority of the visitors onsite at AHF-Checkpoint during the recruitment. Offsite recruitment of end users at events, where a broader range of key populations are tested including heterosexual men and women, was not possible as no testing events were planned between April and June due to the COVID-19 pandemic. Including MSM only was also due to time constraints, as the majority of people who came to test in this period were MSM. Lay providers showed diversity in demographics regarding gender, age, ethnicity, sexual preference, and work experience [23]. All lay providers (n = 10) were invited by the testing and operations manager at AHF-Checkpoint. They were informed by e-mail about the study objectives and asked about their availability for an interview by a researcher from the RIVM. A reminder e-mail was sent after five days. All 10 lay providers participated and were included in the study between April and June 2020.

For the recruitment of end users, a flyer was handed over personally by the testing and operations manager when they visited AHF-Checkpoint for HIV testing. These participants constituted a convenience sample [23]. Due to temporary closure of AHF-Checkpoint for face-to-face activities during 27 March and 11 May (government regulations for contact-based services), and the following restricted opening hours with online planned appointments for testing during the COVID-19 lockdown, the recruitment period was shorter than planned and only twelve end users could be approached for participation in an interview. There were no HIV-positive end users in that period. Eight end users shared an e-mail address or telephone number for sharing study information or to approach them again in case they needed time to consider participation. In case end users did not respond to the e-mail invitation, a reminder e-mail was sent after five days. Three persons did not respond to the first and reminder invitation, resulting in five interviews with end users (response rate 42%). All participants (lay providers and end users) were approached and interviewed by telephone or video calling between April and June 2020. They received a 20 euro gift card as an incentive for participation.

### Data collection and analyses

The interviewer (NK) started the interviews with a short introduction in which the research goals were explained and socio-demographic questions were asked to make participants feel comfortable. An interview guide based on the topic list and open-ended example questions was used to explore the participant’s views on and experiences with the various steps of HIV testing at AHF-Checkpoint.

Lay providers were asked about their reason(s) to volunteer at AHF-Checkpoint, their experiences with the training and guidance they received or needed. They were asked about their views on the counselling and testing process, positive or negative experiences with HIV testing, communication with end users such as the openness to be able to talk about HIV. If lay providers had experience with testing at pop-up locations, their views on the adequacy of those locations was discussed, as well as their views on potential improvements at onsite or pop-up test locations of AHF-Checkpoint.

End users were asked about barriers or facilitators to access HIV testing in general and at AHF-Checkpoint in particular, their views on the counselling and testing process at onsite or pop-up test locations, communication about HIV with the lay provider and in their own social environment, and received guidance in case referral to other healthcare facilities was needed.

During the interview, participants were encouraged to share examples to support their responses, and follow-up questions were used to address any discrepancies in answers [24]. The interviews were audio-recorded and transcribed verbatim in their original language. Data collection continued until data saturation was obtained [25], except for the end-users as inclusion was difficult due to limited opening hours of AHF-Checkpoint caused by COVID-19 restrictions.

The thematic analysis approach of Braun and Clark [26] was followed to derive themes from the answers of lay providers and end users. For reliability and validity, the first two transcripts of each group were analysed by the two RIVM researchers (NK and EO). They developed a coding framework to systematically explore and retrieve insights of different HIV testing modalities, domains and themes. The modalities were (1) testing at AHF-Checkpoint Amsterdam, (2) AHF-Checkpoint pop-up location Rotterdam, and (3) other pop-up testing venues. Domains included emerging themes from the interviews. The WHO 5Cs were specifically asked about in case topics such as confidentiality were not mentioned spontaneously by participants. Views of lay providers and end users on the same topics were compared for validation through data triangulation, to gain an understanding from different perspectives and areas of agreement or divergence. The emerging themes and codes were discussed among the two researchers until consensus was achieved. Quotes of Dutch-speaking participants were translated into English as literally as possible. All quotes were compared with the codes and themes that were identified. Interview data was transcribed using Transcription software HappyScribe and F4 transcript version 7, followed by NVivo analysis software version 10 for thematic analysis.

## Results

### Participants

Of the 15 participants who all consented to be interviewed, ten were lay providers and five were end users of HIV testing at AHF-Checkpoint (Table 1). Twelve out of 15 were male (80%). Seven participants (47%) had a non-Western migration background, of whom five were first-generation migrants, and two were second-generation. Countries of origin were Afghanistan, Colombia, Curacao, Greece, Lebanon, the United States and The Netherlands. Most participants identified as gay (73%) or bisexual (14%). Three lay providers were female. End users were younger (mean age 28.6 years) than lay providers (mean age 35.0 years). Of the lay providers 70% were highly educated, and 20% of the end users. The interviews lasted on average 37 min (range 19–55 min).

**Table 1:** Demographic characteristics of lay providers and end users of HIV testing at AHF-checkpoint

Characteristic	Lay providers (n = 10)	End users (n = 5)	Total (n = 15)
	n (%)	n (%)	n (%)
Sex			
Male	7 (70)	5 (100)	12 (80)
Female	3 (30)	0 (0)	3 (20)
Region of origin			
Western	6 (60)	2 (40)	8 (53)
Non-Western	4 (40)	3 (60)	7 (47)
Age (years)			
< 25	0 (0)	1 (20)	1 (7)
25–39	7 (70)	4 (80)	11 (73)
40–64	3 (30)	0 (0)	3 (20)
Mean (SD) age (years)	35.0 (8.1)	28.6 (6.1)	32.9 (7.9)
Education level			
VET^a^	3 (30)	4 (80)	7 (47)
Higher education^b^	7 (70)	1 (20)	8 (53)
Sexual preference			
Female			
Heterosexual	2 (20)	0 (0)	2 (13)
Bisexual	1 (10)	0 (0)	1 (7)
Male			
Gay	7 (70)	4 (80)	11 (73)
Bisexual	0 (0)	1 (20)	1 (7)

^a^VET: Vocational education and training (Dutch abbreviation ‘MBO’)

^b^Higher education: research-oriented and professional oriented (Dutch abbreviation ‘WO’ and ‘HBO’)

Presented below are the four interrelated domains that emerged from the themes identified from the interviews, namely: (1) Accessibility of HIV testing, (2) Quality of test procedures, (3) Bridging (transitional care), and (4) Future strategies for service delivery. The domains and sub-themes were illustrated by quotes from the participants.

### Accessibility of HIV testing

Lay providers and end users mentioned good accessibility as a key factor for successful HIV testing at onsite and pop-up locations of AHF-Checkpoint. According to lay providers, end users perceive a low threshold to test, such as no barriers like waiting lists or triaging that could include referral to another test location, no testing costs, and quick test results.

Whereas SHCs only provide care for inhabitants of their municipality, AHF-Checkpoint has no restrictions for testing based on age, sexual preference, or postal code area (Table 2, quotes 1–3, lay providers). Although the SHC is a well-known testing facility for HIV and STI, some locations have waiting lists to get tested. When clients experience no symptoms or when they are not considered ‘high risk’, the waiting time can be several weeks (quote 2, lay provider). Most lay providers reported that end users are often amazed by the fast service and the same-day result at AHF-Checkpoint. The importance of having no waiting lists and the rapid result were also pointed out by end users (quotes 4–5). For some end users having had a positive previous experience at AHF-Checkpoint was a reason to return. End users appreciated the discreet environment of AHF-Checkpoint and its convenient location: above a second-hand clothing store in the city centre of Amsterdam (quotes 4–6, end users). However, one end user mentioned that it was not so easy to find (quote 6).

**Table 2:** Quotes associated with the four domains from the interviews with lay providers and end users of HIV testing at AHF-Checkpoint

1. Accessibility of HIV testing	
1	So, we do not give priority to someone. Of course, we encourage MSM to test, because of their high risk. But we test everyone, we test heterosexuals, women with a very, very, very low risk. So, we do not say no to anyone. We test every person and that is what I like about AHF [Checkpoint] [Lay provider 4, MSM, 26 years]
2	At the sexual health clinic you really have to fill out a very long form before you even can get an appointment. And if you get an appointment, it takes really three or four weeks [Lay provider 6, MSM, 38 years]
3	They [SHC] are working from eight to four, and I can never reach them again. Why is not there a sexual health clinic open on Saturday for HIV testing or STI testing? … They are very strict when you come from a different zip code area … clients were annoyed [Lay provider 3, HIV infected, 52 years]
4	Well, first of all it was near, I live close so that was good. Second, it's for free, so that’s a very plus. Third, it’s anonymous … I did not have to sign up … It was more like, you go there, you wait and then you get tested. … those were the three key aspects I would say [End user 3, MSM, 31 years]
5	.. you will hear the result quickly. And two, you do not have to pay a deductible. It is free. And three, if you just test… then the stress just immediately decreases [End user 4, MSM, 22 years]
6	To be true, it was not super easy to find, because it was kind of sketchy, you need to go into a closet store and then go upstairs. But once you are there.. at the same time, it is also kind of convenient, because, it is not that everybody can see that you are entering a testing facility for HIV … Although, we should not be ashamed of that [End user 3, MSM, 31 years]
7	And that was within four or five text messages, we had arranged an appointment. I really liked it! [End user 1, MSM, 26 years]
8	Because everyone is living in AZC [centre for asylum seekers] and we [refugees] do not have all the knowledge about the health care in The Netherlands. So, they wait in line… and half of them are MSM or transgender who are having sex with men. So, they wait ever since to test [for HIV]. So that's why my first testing experience with AHF was pretty good. I tested a lot, 60 tests! [Lay provider 4, MSM, former refugee, 26 years]
9	So, it’s a different kind of people, they are mostly heterosexual and another community. So, they are not interested. You see the differences when you test at a gay festival or a queer festival. … You see another reaction ‘No I’m clean, no I’m clean’. So, they have this thing that they are clean [Lay provider 4, MSM, 26 years]
2. Quality of test procedures	
10	When they do not live in Amsterdam or are not insured, they are afraid, or they have no documents and are afraid that they will come into contact with the immigration police … People are.. they feel safe with us … if they are inside Checkpoint it is already a victory.” [Lay provider 2, MSM, 32 years]
11	From my experience working with African populations abroad, I do understand the cultural barriers … The way they like to do things.. like the process that involves with gaining their trust and making sure you do things the right way before you even think about talking about testing with them [Lay provider 1, female, 40 years]
12	.. a strong feature is just listening. Listen more. We want to help so badly and then we go straight to the help mode. But by just listening to the customer and listen to his story, “What happened?”, then you know exactly what kind of approach needs to be done [Lay provider 6, MSM, 38 years]
13	[Addressing a panicked client] So, I start to relax them, I change the subject. I talk about the weather, I talk about pets. Anything else, just to make them ready for the test, because at Checkpoint it is not that busy. We have time to communicate with the client. This is a good thing! You can give him or her all the info they want [Lay provider 4, MSM, 26 years]
14	It is not a subject that I discuss with friends or family. It is not that it has a stigma, but it is not that you are talking about it quickly. So, no, I would not know how they would react if I started talking about HIV testing. I have no idea [End user 5, MSM, 26 years]
15	They always explain me first how the test works. … how you should read the result. Then they show me the results. So basically, I am part of the test! … and I can see the test working. So that, uh, gives more confidence [End user 3, MSM, 31 years]
16	When I’m at Checkpoint … They (lay providers) should say “Okay now, you do not have HIV. By the way, we also recommend that you have a regular test on other STI's if you want. It is recommended. And these are places were you also could go in case you are interested”. But at least, OPEN THE DOOR! (to test for other STI’s) [End user 3, MSM, 31 years]
3. Bridging (transitional care)	
17	So, if I’m taking the shift on Saturday and someone comes with a reactive result. So, I need to take his info and tell him that on Monday someone will call you. And I do not like this thing, to be honest. Because we cannot refer on Saturday. Everything is closed. When someone has a reactive result, I do not think he can wait until Monday for support. It’s two days! [Lay provider 4, MSM, 26 years]
18	If we just, kind of stop focusing. ‘I am my organisation AHF, you are PHS’. So, everyone does their own thing. We all have the same purpose, the same goal. So why not just work together and achieve what they all want? Together we are obviously stronger! [Lay provider 6, MSM, 38 years]
19	So in Amsterdam, if someone comes to the Checkpoint and tested positive, we can send them to one place if they do not have insurance or if they're a refugee, or if they do have insurance we send them somewhere else … the lines are a little more clear. But when you're somewhere else (off-site testing), it's less familiar. You're not familiar with the event and the clientele. And then after the event and after the test where you can refer those people to? … I want a.. a liaison! [Lay provider 9, MSM, 26 years]
4. Future strategies for service delivery	
20	I’m assuming they [AHF-Checkpoint] already do that, but I think they might advertise even more with the fact that they exist and that you have the possibility to test. Maybe via social media too, (because) I know exactly how to look it up online and stuff, but it could be easier or more user-friendly [End user 2, MSM, 38 years]
21	So that you no longer have to physically go to a place for a training, but that you develop a number of training courses yourself and that you can view them once in a while (E-learning)... Or make your own document, reference book, as an organization. I think it’s just important that you just make sure you have your own information available [Lay provider 7, MSM, 27 years]
22	I have no idea of what are the symptoms for chlamydia or what are the symptoms for syphilis or any of the other STI's. I know, sort of.. when I was taught at school. But that is (a) long time ago [End user 3, MSM, 31 years]
23	If you're going to do everything [STI testing], you’re not going to be successful. You have to be specific … One thing only and people who look for you, for one thing only … So it's better to be good in HIV testing, than bad in testing for all [STI] [Lay provider 4, MSM, 26 years]
24	AHF, based on my experience, really only wants to test positive people. So they go looking for the people in the MSM-community. While I'm looking for the one or two people in the straight community who are also positive. But AHF says no! We have to cut costs, so we need positives, so we go more to the MSM-community… That's where we find most [HIV positive people] [Lay provider 3, MSM, 52 years]

Scheduling an appointment at AHF-Checkpoint via an online platform and text messaging during the COVID-19 pandemic appeared sufficient (quote 7, end user). This appointment option was added in order to better plan and continue providing care at AHF-Checkpoint during the COVID-19 lockdown when opening hours were restricted.

At pop-up locations, AHF-Checkpoint aims to test as many visitors as possible. Some events were very successful according to lay providers, especially events for LGBTQ refugees, where up to 60–100 visitors are tested in one day and people are lining up for an HIV test (quote 8, lay provider). However, reaching heterosexual people at events seems more difficult than reaching MSM. Heterosexuals may have different perceptions of personal risk for HIV infection or attitudes toward HIV testing (quote 9, lay provider).

### Quality of test procedures

Confidentiality and anonymity were guaranteed at the test location in Amsterdam and the onsite pop-up location in Rotterdam. Lay providers explained that onsite testing has some advantages compared to HIV testing with the mobile unit (testing van) at events, where “*anyone can see you coming in and out”* (lay provider 9). At events, such as refugee nights, people queue in front of the testing van. Lay providers reported that fear of being seen by others could induce a barrier to test at such an event. This issue was only addressed by lay providers. Only one of the end users was tested at an outreach setting and this was not a mobile unit but a private room at a nightclub, where confidentiality may not have been a problem.

It is important that end users, especially if they are refugees, feel safe at an HIV test location (quote 10, lay provider). If lay providers have similar cultural backgrounds or having HIV, this may encourage open discussions with end users about HIV testing. They could easily show empathy and relate to the end users (‘levelling’), as they themselves may have experienced stigma or fear for testing in countries where they have lived (quote 11, lay provider). Being discreet, friendly, and non-judgmental are important characteristics for lay providers in order to build a trustful relation with end users, and being a good listener could make them feel at ease (quotes 11–13, lay providers). End users appreciated the fact that they could communicate openly at AHF-Checkpoint about their sexuality and reason(s) to test. Not all end users could talk openly about HIV or testing with friends or other social contacts (quote 14, end user).

After verbal consent, lay providers always discuss their clients’ risk with an intake questionnaire, also in relation to the window phase of the rapid test. Clients are asked to return at a later date for testing when the risk was less than 6–8 weeks ago. Lay providers reported that they follow the counselling procedures strictly, such as informing clients about the steps of the HIV rapid test, discuss their (sexual) risk behaviour, and reserving time for questions. All lay providers felt competent to conduct these steps. End users experienced the explanation of each step as important to gain trust and involvement in a correct test result. One of the end users described it as ‘feeling part of the procedure’ (quote 15, end user).

Although all lay providers were trained, some felt unprepared for emotionally charged experiences, such as communicating an HIV-positive result or being able to deal with unexpected situations in different ways (tailoring). Some expressed a need for culturally and linguistically appropriate communication tools around sexual health. However, AHF-Checkpoint supervisors are always reachable to provide support when needed or to evaluate a difficult counselling session afterwards. This support and guidance increased their confidence to follow procedures correctly. No perceived barriers or negative experiences during HIV testing and counselling were reported by the end users, but the number of end users included in the study was limited. One end user expressed a need for more information on where to test for other STI (quote 16, end user).

### Bridging (transitional care)

A well-founded bridge for HIV confirmation testing and STI testing should be built between AHF-Checkpoint, SHC, GPs and hospitals, as suggested by some lay providers and end users (quotes 16–19). When an HIV test is reactive at AHF-Checkpoint, another test of a different brand is carried out to minimize the risk of a false positive result. In a next step, end users are send to one of the HIV treatment clinics or the SHC for confirmation testing and treatment. Follow-up for confirmation testing was improved in the past years according to lay providers. Now, AHF-Checkpoint obtains clients’ consent to contact them within a month to ‘check-in’ whether they had been successfully linked to care (connection to prevention and care). On average, 80% of the end users with a reactive test are successfully referred. However, tourists and expats may arrange their own follow-up in their country of origin, so connection-to-care is likely higher than the reported 80% [19]. Nevertheless, some lay providers acknowledged that referral-to-care on weekend days was occasionally difficult, leading to an inconsistent level of support as SHCs or collaborating hospitals can be reached till 4 p.m. on Saturday for a confirmation test. Thus, end users with a reactive result then remain in uncertainty until Monday (quote 17, lay provider). To secure confirmation testing, also for pop-up locations, a closer collaboration with other healthcare facilities is needed (quote 19, lay provider). Having a liaison for HIV care as well as for STI testing with local professional healthcare providers was recommended by lay providers as well as end users (quotes 18–19).

### Future strategies for service delivery

To improve community-based HIV testing at AHF-Checkpoint in the future recommendations were made by lay providers and end users, such as extra publicity to increase awareness about HIV testing at AHF-Checkpoint through social media (quote 20, end user). Culturally and linguistically appropriate communication tools on sexual health were suggested. One person expressed a need for a reference work on sexual health, for instance an e-learning tool to improve general knowledge on STIs and on cultural differences regarding STI, or to develop a reference work themselves (quote 21, lay provider). End users also reported having lack of knowledge on symptoms of STI (quote 22, end user). However, opinions on having both HIV and STI testing at AHF-Checkpoint varied between participants. Some lay providers and end users recommended testing for HIV only at AHF-Checkpoint, as “*it’s better to be good in HIV testing, than bad in testing for all [STI]*” (quote 23, lay provider)***.***

From its conception, AHF-Checkpoint focused on MSM populations, migrants including heterosexual men, women, and transgender persons. Yet, with their outreach activities, they make a strong effort to reach high-risk populations including LGBTQ communities and refugees. These choices are partly driven by the budget that AHF-Checkpoint receives from AHF Europe as the program aims to be effective, cost-effective and reach certain positivity rate. For AHF-Checkpoint in The Netherlands this goal is a one percent positivity. To a certain level this complicates the efforts to find the undiagnosed among populations at lower HIV risk (quote 24, lay provider), who are more dispersed and thus more difficult to reach. However, AHF-Checkpoint recently extended their activities to heterosexual populations in multicultural areas such as ‘Amsterdam-Southeast’ where the testing van visits once a month. This offers women, illegal immigrants and people with lower incomes an opportunity to test without tests or travel costs. Thus, the AHF-Checkpoint team plans its activities and focus based on the epidemiological situation and geolocation as testing needs to be effective, meaning certain planned targets related to number of tests and positivity need to be reached.

## Discussion

The community-based HIV test facility AHF-Checkpoint fills a gap for people who experience barriers to HIV testing at regular test sites such as SHCs or GPs, as was illustrated by both lay providers and end users. The free HIV test service with same day results was much appreciated by the end users, as well as the friendly and trustful contact with the lay providers. Motivations of end users to choose a community-based test service over a regular healthcare setting were mostly practical: avoiding waiting lists and getting a quick result, or having had a positive previous experience at AHF-Checkpoint. More complex issues as described in (inter)national literature [27, 28], such as fear for stigma and discrimination, lack of confidentiality in governmental institutions, or test costs at the GP were not reported as motivations to test at AHF-Checkpoint.

We evaluated AHF-Checkpoint in the context of the WHO 5Cs framework of rights-based response to HIV testing. Consent, counselling, and correct test results were all established, but confidentiality was sometimes challenging at outreach locations where the mobile unit was used, and quick referral for confirmation testing during weekends was sometimes lacking (connection-to-care). Similar shortcomings of the 5Cs principle were reported in a Spanish study on provider-initiated HIV testing that included community-based test sites [29], and in a systematic review on HIV testing strategies outside regular healthcare settings [16].

Although end users at AHF-Checkpoint were self-aware about their sexual risks, there was an apparent need for education on STIs for end users as well as lay providers, but not necessarily a need for STI testing at AHF-Checkpoint. Educational activities on STI for lay providers could support better knowledge, which in turn would positively influence the quality of counselling on STI for end users [3, 19, 30]. STI testing could be arranged by a liaison approach with professionals from regular test sites having consultations at AHF-Checkpoint. An Australian study on a non-clinical HIV service testing called, PRONTO! showed that end users seemed to prefer doctors or nurses when compared to lay providers [31]. This may indicate that lay providers’ involvement is accepted by clients, but does not have to be the decisive factor to choose for AHF-Checkpoint. Just like the recent rapid syphilis testing pilot at AHF-Checkpoint, PRONTO! attempted to combine STI testing with HIV testing but concluded that it was not feasible without sustainable funding [31]. A study conducted among MSM at a checkpoint in Barcelona showed a high incidence of STI, but also a lack of retesting for STI when the lab result was indeterminate or inconclusive [32]. Although combined HIV and STI testing at community sites sounds attractive, economic and logistic restraints were reported. Yet, closer collaboration with local SHCs could be considered for community-based services as AHF-Checkpoint to subsequently guide key populations into broader STI/HIV care [33].

There are some study limitations to report. First, data saturation was only reached for lay providers. The recruitment of end users was hampered by the COVID-19 pandemic and government restrictions on opening hours of the service. Second, the sample only included end users who were all MSM and had a negative HIV test result. Participants from other key populations or those with a reactive test result may have had a different view. Nonetheless, the sampling resulted in a rich dataset with a broad range of perspectives from participants with various migration backgrounds, including former refugees, a lay provider with HIV, and lay providers with different years of testing experience. [34] Furthermore, the interviewer has tried to establish a safe atmosphere with empathy to gain access to the participants’ stories, which could have reduced potential bias, as the ambiance at all interviews was open and confidential [35].

Although AHF-Checkpoint may reach a different target group than SHCs, as the proportion of first time testers is higher (26%, mostly MSM) compared to SHCs (11% among MSM) [19, 20], it is doubtful that a single test service with a limited capacity will have a significant population-level impact on HIV transmission in The Netherlands, but a service model that is replicated might have. The ability of having population-level impact is connected with the overall budget of AHF-Checkpoint that influences the workforce and the geographical locations of the services. While expanding their services or pop-up activities might not be feasible at short-term due to limited budgets and the COVID-19 pandemic, improvements without extra costs are still possible. Lay providers indicated a need for e-learning, next to their group- or face-to-face sessions to better prepare for emotionally charged situations and a (culturally) tailored approach. STI Aids Netherlands developed various e-learning modules (‘digitale leerweken’ https://leren.soaaids.nl/) on STIs, HIV care, motivational interviewing, partner notification, chemsex, PrEP, and U = U (undetectable = untransmittable) that can be followed for free.

Improvements of connection-to-care and extra publicity on social media are needed to get more people tested and referred to care. Referral for confirmation testing of those with an HIV reactive test was an occasional challenge during weekends, and solutions must be sought with local healthcare professionals. Furthermore, STI consultation hours at AHF-Checkpoint by local professionals should be considered, but this will lead to extra costs. Finally, implementation of SMS reminders were recommended to encourage repeat HIV testing at AHF-Checkpoint. End users seem to be willing to share a phone number, as they also did to make a test appointment during the COVID-19 lockdown. So this platform can be used for follow-up of confirmation testing and linkage to care, which according to the WHO is less costly than in-person support mechanisms [36].

## Conclusion

AHF-Checkpoint was described as a convenient and easily accessible service by end users and lay providers. Of the WHO 5Cs, connection-to-care could be optimised to ensure HIV confirmation and STI testing through a liaison approach with professionals from the regular healthcare sector. The community based HIV testing service AHF-Checkpoint offers testing to everyone who needs it, but reaches mainly high risk populations such as MSM and migrant populations. Budget limitations and the need to be effective in terms of planned targets related to number of tests and positivity may get in conflict with their goal to be inclusive and offer testing without restrictions.

## Data Availability

The qualitative manuscripts analysed during the current study are not publicly available due to privacy concerns of those interviewed. The datasets generated and analysed are available through the corresponding author on reasonable request.
